# Teaching high school students to use online consumer health resources on mobile phones: outcome of a pilot project in Oyo State, Nigeria

**DOI:** 10.5195/jmla.2019.536

**Published:** 2019-04-01

**Authors:** Grace Ada Ajuwon, Ademola Johnson Ajuwon

**Affiliations:** Principal Librarian, Users Services, E. Latunde Odeku Medical Library, College of Medicine, University of Ibadan, Ibadan, Nigeria, gajuwon@com.ui.edu.ng; Professor, Department of Health Promotion and Education, Faculty of Public Health, College of Medicine, University of Ibadan, Ibadan, Nigeria, ajajuwon@yahoo.com

## Abstract

**Objective:**

This project evaluated the outcomes of training high school students to deliver consumer health information to their peers.

**Methods:**

A total of 120 students selected from 7 high schools in Oyo state, Nigeria, received 8 hours of training on consumer health literacy and peer education, which is a process of training volunteers to deliver health information to their peers. The training included hands-on activities using the students’ own mobile phones. After the training, peer educators distributed leaflets, showed consumer health information (CHI) websites to others, counseled and referred fellow students, and submitted forms describing these activities. All peer educators completed pre- and post-tests, and 10 were interviewed 4 months after training.

**Results:**

After the training, the authors found improvement in the trainees’ knowledge of CHI resources and understanding of their roles as peer educators. Most peer educators (72.5%) delivered CHI to their peers after the training, primarily through sharing websites on teen health and other CHI resources. In the interviews, all peer educators reported direct benefits from participating in the project, and many stated that they knew where to find reliable health information.

**Conclusion:**

Volunteer high school students can be trained to deliver CHI to their peers using mobile phones.

## INTRODUCTION

The advent of the Internet has improved access to health information globally. It has raised awareness of various types of health information that enable individuals to be actively involved in their own health care decision making. During the last decade, health literacy has become a key topical issue as more people play important roles in the care and management of their own health.

The Medical Library Association (MLA) has defined health literacy as “the set of abilities needed to recognize a health information need; identify likely information sources and use them to retrieve relevant information; assess the quality of the information and its applicability to a specific situation; and analyze, understand, and use the information to make good health decisions” [1–5]. An early definition of health literacy is “cognitive and social skills which determine the motivation and ability of individuals to gain access to, understand and use information in ways which promote and maintain good health” [6]. Health literacy has also been defined as “the degree to which individuals have the capacity to obtain, process, and understand basic health information and services needed to make appropriate health decisions” [7, 8]. Health literacy requires that individuals know where to locate and how to act on credible information to answer health questions [9].

Consumer health information (CHI) refers to information that professionals deliver in response to requests from their clients or the general public, including patients and their family members [10]. The importance of CHI is underscored by the fact that it is beginning to be reflected in health policies in some countries, such as the United Kingdom [11], with implications for information producers, providers, and users and a recommendation to advance more patient-centered health services [12]. A large quantity of CHI is available through Internet search engines, meaning that health care consumers increasingly have more opportunities to obtain information regarding all aspects of health care largely due to improved access to electronic health information [13]. The vast volume of health information available on the Internet requires users to develop literacy skills that include the ability to carefully filter and select this information.

However, many users are also turning to public and hospital libraries to access current, credible, and comprehensive health information that is available in these settings [14]. Two problems have emerged in this process. On the one hand, consumers often do not know how to evaluate health information in either print or electronic format, whereas on the other hand, public librarians have difficulty evaluating and knowing where to find reliable health information [15–18]. Many librarians are uncomfortable responding to health questions due to lack of knowledge or because they are unsure how to interact with patrons who are requesting such assistance [19]. Some patrons have also shown displeasure with their encounters with public librarians when they requested health information [20].

Still, libraries and librarians have made important contributions through health literacy teaching, research, services, and programming [21]. Some of these efforts involve educational interventions and outreach programs [22]. Libraries have created educational programs, materials, online tutorials, and tool kits for health workers [23–25]; partnered with health and community organizations; and used innovative methods to reach their clients such as aged or underserved populations, health care professionals, students, patients, and the general public [21]. Libraries have also collaborated with other organizations to promote CHI literacy by targeting different populations, including adolescents [22–27].

Adolescents are an important target of consumer health literacy programs for three reasons. First, adolescence is a formative and highly relevant period for behavior development. The information that young people acquire during this period affects their health in adulthood. Second, health literacy programming for adolescents is aimed at preventing diseases, including sexually transmitted infections and mental disorders such as depression, to which many adolescents are vulnerable as they navigate the transition into adulthood [23, 28, 29]. Finally, many adolescents access the Internet for health information [9, 24, 30–32], which creates novel opportunities to use this channel to reach this group for health interventions.

Although the Internet is an important source of health information, not all materials available online are credible. As a result, adolescents require literacy skills to carefully filter and select the vast volume of online health information. Adolescents also need formal training to search, locate, assess, retrieve, evaluate, understand, and use information that is available on the Internet to make informed decisions about their health [33].

Although consumer health literacy interventions are available in Nigeria [10], few have focused on adolescents. The project described in this article is a consumer health literacy program that targeted high school students in Oyo state, Nigeria, using peer education, a process by which trained persons teach or share health information and promote healthy behavior among peers with whom they share similar social characteristics. This approach is supported by research showing that people are more likely to receive messages, personalize the messages, and change their attitudes and behaviors if the messenger is similar to them and shares the same concerns and pressures [34]. Peer education is considered appropriate for consumer health literacy among high school students because it draws on the credibility that young people have among their peers. Moreover, it leverages the power of role modeling and flexibility in meeting consumer health information needs of adolescents [34].

## METHODS

### Setting

This project was conducted between 2016 and 2017 in the Ibadan metropolis and the town of Oyo. The population of Oyo state is approximately five million people who are mainly Yoruba, the major ethnic group in southwestern Nigeria. The study population consisted of adolescents enrolled in public and private high schools. In Nigeria, high school education consists of junior (JSS1–JSS3) and senior (SSS1–SSS3) classes, each with a duration of three years of classroom instruction covering the arts, sciences, and social sciences. Mobile phones were readily available in Oyo state in 2016–2017 when the study was conducted. The widespread ownership and use of mobile phones in Nigeria [35] provided an excellent opportunity to use these devices in the delivery of a consumer health literacy intervention.

### Project phases

High school students were recruited and trained, after which they were expected to discuss and inform their peers about health literacy using their personal mobile phones. They were also expected to distribute leaflets about health literacy and keep a record of their activities. The project was implemented in three phases: planning, intervention, and post-intervention.

#### Planning

The authors selected and visited seven high schools in the study areas to obtain permission and support for the project from the administrators. At our request, each school selected a teacher who worked with the investigating team to plan the logistics of the project, including the recruitment of students, dates, content, and venue of training.

The schools nominated students to be trained based on our criteria: suitable candidates needed to be regular students who owned a mobile phone and who were willing to participate. Because students were not allowed to use mobile phones on school premises, the administrators gave permission for nominated students to bring their own mobile phones to school, as they were needed for hands-on activities. We designed and produced a leaflet (supplemental Appendix A), a printed sheet of paper distributed by hand containing web addresses and other relevant CHI; a pre- and post-test questionnaire (supplemental Appendix B); a peer educator (PE) activity form containing the name of the PE, school, gender, and date of educational activity (supplemental Appendix C); and an interview guide (supplemental Appendix D).

#### Intervention

A total of 120 nominated students received 8 hours of CHI training as PEs. The training took place in 4 cohorts (30 students/cohort) inside a classroom in a participating school between October 2016 and January 2017. The objective of the training was to empower PEs with knowledge and skills that would enable them to serve as peer promoters of CHI in their schools.

The content of the training was definitions of consumer health, online sources of CHI, and peer education. These were delivered through lectures, discussions, and hands-on activities that included where to access accurate and quality health information and browsing of specific websites where adolescents could access reliable CHI that was relevant to their needs (e.g., TeensHealth, KidsHealth, the Government of South Australia’s Women’s and Children’s Health Network: Child and Youth Health, the US National Library of Medicine’s MedlinePlus, the US Center for Disease Control and Prevention’s Adolescent and School Health, Info Health, and the Nemours Foundation: Children’s Health System).

The PEs received mobile Internet data bundles on their own mobile phones before commencing the training to enable them to fully participate in hands-on activities. They also completed the pre- and post-test questionnaire to assess immediate training outcomes. One teacher from each school observed the training process and was subsequently requested to supervise PE activities. Investigators provided each PE with twenty leaflets and copies of the PE activity form.

The health sciences librarian educator introduced the concept of health literacy and taught PEs how to launch the browsers on their phones and connect to the Internet to access online consumer health resources. The librarian also taught the students how to navigate each website. The health promotion expert defined the meaning of peer education and identified benefits of peer education, attributes of a good PE, and roles of a PE in a consumer health literacy program in a school setting. This joint facilitation of training sessions created synergy aimed at improving the PEs’ understanding of consumer health and showing them how to deliver this intervention to their peers.

#### Post-intervention assessment

Four months after training, we randomly selected 14 PEs (2 from each of the 7 schools that participated in the project) for face-to-face interviews. Ten (71.4%) of the 14 selected PEs consented to be interviewed. The purpose of the interviews was to document the PEs’ perceived usefulness of the training, their peers’ reception of the activities, challenges they encountered, and their suggestions for improvement. The interviews were recorded on audiotapes after respondents provided consent.

### Data management and analysis

All quantitative data were collated, entered into a computer, and analyzed using SPSS software version 15. Pre- and post-test data were compared using chi-squared tests, with *p*<0.05 considered statistically significant. Data from the PE activity forms were collated, summarized, and presented using simple percentages. Qualitative data from the audiotaped recordings were transcribed and subjected to thematic analysis in addition to representative verbatim quotations.

## RESULTS

### Pre- and post-tests

Equal numbers of PEs were from private and public schools (Table 1). The mean age of PEs was 15.5 years (standard deviation, 1.6). There were more male (54.2%) than female (45.8%) PEs.

**Table 1:**
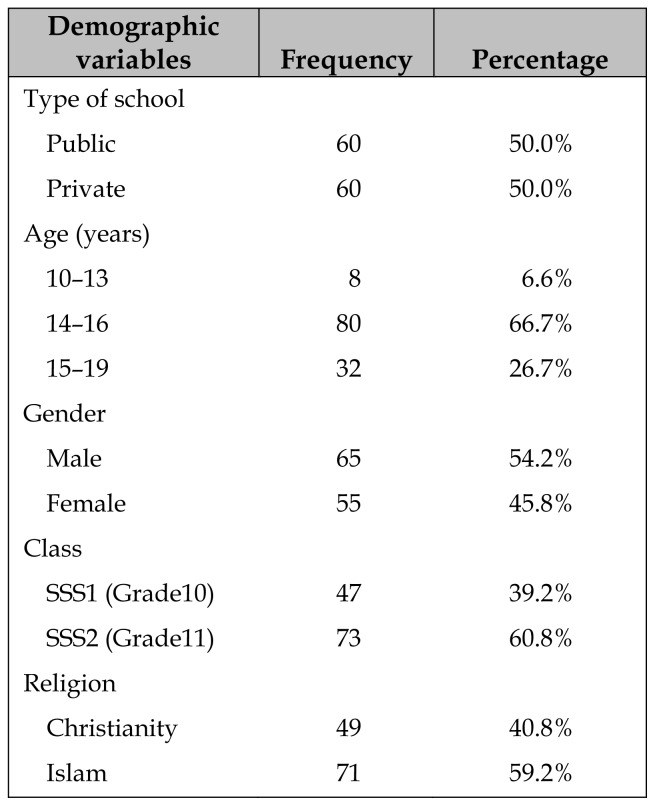
Demographic profile of peer educator (PEs) in Nigeria (n=120)

Demographic variables	Frequency	Percentage
Type of school		
Public	60	50.0%
Private	60	50.0%
Age (years)		
10–13	8	6.6%
14–16	80	66.7%
15–19	32	26.7%
Gender		
Male	65	54.2%
Female	55	45.8%
Class		
SSS1 (Grade10)	47	39.2%
SSS2 (Grade11)	73	60.8%
Religion		
Christianity	49	40.8%
Islam	71	59.2%

Only 23.3% of PEs had heard about CHI before the training, which increased to 90.1% after the training (Table 2). Before the training, PEs listed several health information sources they knew, with Google (13.1%), Yahoo (8.4%), and television (7.9%) being the most well-known sources; 41.9% did not respond to the question. After training, however, PEs were more likely to list credible health information sources such as TeensHealth (19.2%), Info Health (15.4%), and MedlinePlus (13.5%). Whereas PEs consulted a variety of health information resources before training, including Google (21.0%), Yahoo (14.3%), and Facebook (17.3%); after the training, PEs were more likely to consult credible CHI sources including TeensHealth/KidsHealth (24.5%), MedlinePlus (17.9%), and Info Health (11.5%).

**Table 2:**
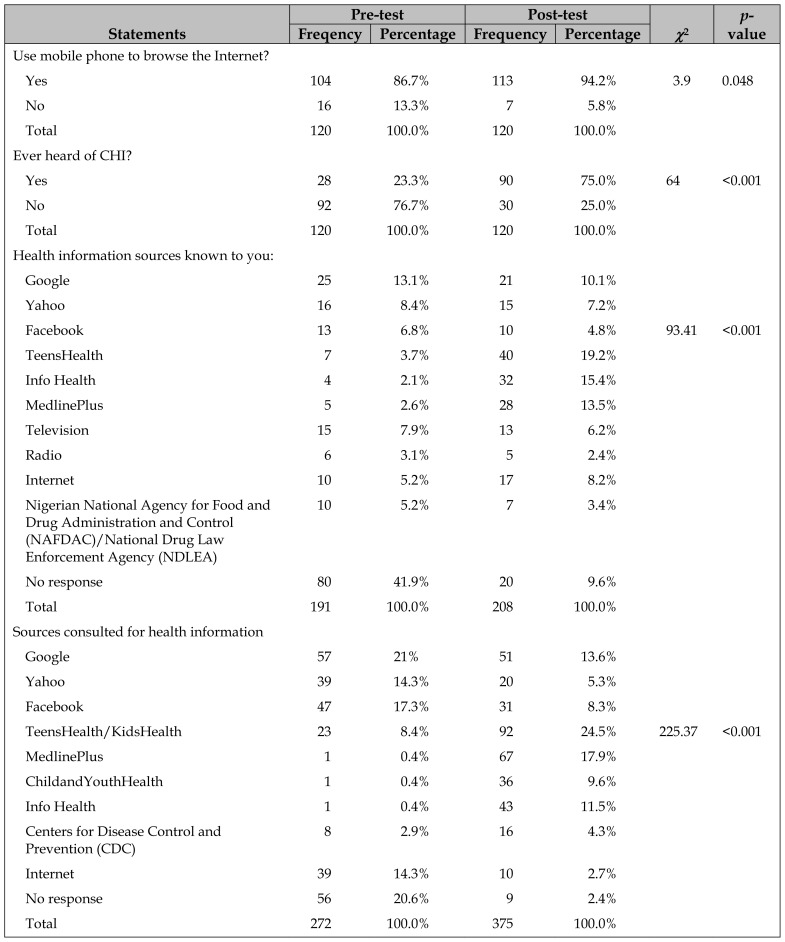
Percentage of PEs who were aware of consumer health information (CHI) and CHI resources

Statements	Pre-test		Post-test		*χ*^2^	*p*-value

	Freqency	Percentage	Frequency	Percentage		
Use mobile phone to browse the Internet?						
Yes	104	86.7%	113	94.2%	3.9	0.048
No	16	13.3%	7	5.8%		
Total	120	100.0%	120	100.0%		
Ever heard of CHI?						
Yes	28	23.3%	90	75.0%	64	<0.001
No	92	76.7%	30	25.0%		
Total	120	100.0%	120	100.0%		
Health information sources known to you:						
Google	25	13.1%	21	10.1%		
Yahoo	16	8.4%	15	7.2%		
Facebook	13	6.8%	10	4.8%	93.41	<0.001
TeensHealth	7	3.7%	40	19.2%		
Info Health	4	2.1%	32	15.4%		
MedlinePlus	5	2.6%	28	13.5%		
Television	15	7.9%	13	6.2%		
Radio	6	3.1%	5	2.4%		
Internet	10	5.2%	17	8.2%		
Nigerian National Agency for Food and Drug Administration and Control (NAFDAC)/National Drug Law Enforcement Agency (NDLEA)	10	5.2%	7	3.4%		
No response	80	41.9%	20	9.6%		
Total	191	100.0%	208	100.0%		
Sources consulted for health information						
Google	57	21%	51	13.6%		
Yahoo	39	14.3%	20	5.3%		
Facebook	47	17.3%	31	8.3%		
TeensHealth/KidsHealth	23	8.4%	92	24.5%	225.37	<0.001
MedlinePlus	1	0.4%	67	17.9%		
ChildandYouthHealth	1	0.4%	36	9.6%		
Info Health	1	0.4%	43	11.5%		
Centers for Disease Control and Prevention (CDC)	8	2.9%	16	4.3%		
Internet	39	14.3%	10	2.7%		
No response	56	20.6%	9	2.4%		
Total	272	100.0%	375	100.0%		

Only 37.5% of PEs correctly defined a peer educator before training, whereas 87.5% did so after (Table 3). In addition, 27.5% of PEs could list at least 1 quality of a good PE before training, where 99.2% could do so after.

**Table 3:**
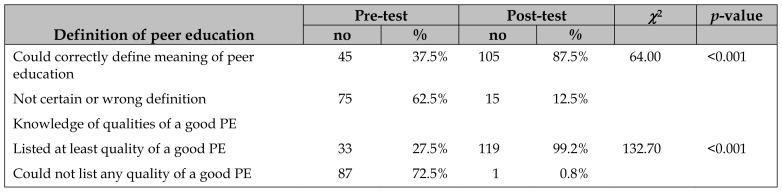
Knowledge of definition and qualities of a good PE (n=120)

Definition of peer education	Pre-test		Post-test		*χ*^2^	*p*-value

	no	%	no	%		
Could correctly define meaning of peer education	45	37.5%	105	87.5%	64.00	<0.001
Not certain or wrong definition	75	62.5%	15	12.5%		
Knowledge of qualities of a good PE						
Listed at least quality of a good PE	33	27.5%	119	99.2%	132.70	<0.001
Could not list any quality of a good PE	87	72.5%	1	0.8%		

### Post-training activities

The PEs commenced consumer health promotional activities immediately after training. We conducted 2 post-training evaluations: collection of PE activity forms and interviews with the PEs. We collected completed PE activity forms from the teachers every 2 weeks for 4 months after training. We found that of the 120 PEs trained, 87 (72.5%) submitted an activity form; most PEs (81.6%) submitted only 1 form, whereas some (18.4%) submitted more than 1 form. The maximum number of forms submitted by a single PE was 3. The PEs most frequently engaged in showing CHI websites to their peers, followed by counseling, distributing leaflets, and referring peers to a public librarian or health care provider (Table 4). We found no significant differences in engagement in these activities between male and female PEs or between PEs from private versus public schools.

**Table 4:**
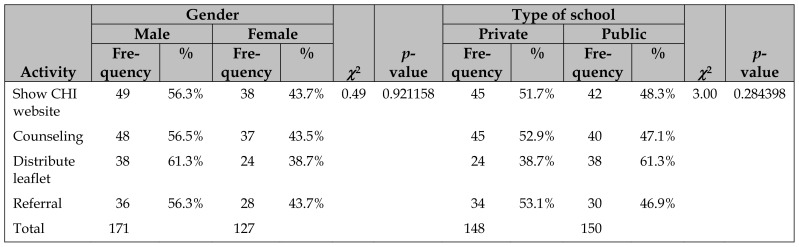
Summary of PE activities by gender and type of school

Activity	Gender				*χ*^2^	*p*-value	Type of school				*χ*^2^	*p*-value
	
	Male		Female				Private		Public			
	
	Frequency	%	Frequency	%			Frequency	%	Frequency	%		
Show CHI website	49	56.3%	38	43.7%	0.49	0.921158	45	51.7%	42	48.3%	3.00	0.284398
Counseling	48	56.5%	37	43.5%			45	52.9%	40	47.1%		
Distribute leaflet	38	61.3%	24	38.7%			24	38.7%	38	61.3%		
Referral	36	56.3%	28	43.7%			34	53.1%	30	46.9%		
Total	171		127				148		150			

### Post-intervention interviews

#### Perceived usefulness of training

There was consensus among the PEs that the training was useful to them. As one PE put it: “I now know where to get information to take care of my health.” Another illustrative response was: “they gave us websites which I use to go to get information for my own personal health knowledge.”

#### Activities performed

All interviewed PEs reported that they had performed their roles as consumer health promoters to their peers and others. For example, one PE stated, “I have been distributing leaflets, showing the websites, and counseling them [peers].” Counseling was the most frequently mentioned activity that the PEs performed, followed by showing websites. As one PE explained, “I enjoyed counseling [without the phone] most because it does not require much effort.” The conversation below also illustrates this point:

Interviewer: When did you last provide CHI to someone?

Interviewee: I did for my mother yesterday.

Interviewer: How did you do it?

Interviewee: She talked about having a headache and there was one leaflet with me. When I went through the leaflet, I found some possible solutions: that maybe she is thinking too much, and she said “yes.” That is one of the causes of headache when we searched (the Internet); I then advised her to go to the hospital.

The PEs reported that many people they counseled received the information well. As a result, some of those who were counseled stated that they desired to be a PE in the future because PEs were regarded as “health information providers” and “gained more recognition than before.”

#### Challenges encountered

The PEs encountered three problems when engaging in CHI activities. One problem related to showing websites and was attributed to poor telephone network connectivity, which “[made] it difficult to teach others.” Another problem was that PEs were not given sufficient number of leaflets. As a result, the PEs said that it was “difficult for people to believe me if they [did] not see the leaflet.” A final problem with respect to counseling was related to discussing sensitive topics like sexual intercourse.

#### Suggestions for improving future training

The PEs offered four suggestions for improving future training: (1) training new students and retraining PEs; (2) using social media, such as Facebook and Twitter, to disseminate CHI to students; (3) providing students with free mobile phones during training rather than asking them to bring their own for hands-on activities; and (4) video-recording a training session to be later shown to students who did not have the opportunity to be trained.

## DISCUSSION

Our results indicate that it is feasible to conduct a school-based consumer health literacy program targeting adolescent students using mobile phones. To our knowledge, this is one of the first consumer health literacy intervention projects in Nigeria that was jointly implemented by a health sciences librarian, a public librarian, and a health promotion professional in partnership with high school teachers and school administrators targeting in-school adolescents. This project is innovative due to its peer-to-peer nature, using adolescents themselves to deliver CHI in high school settings. The introduction of mobile connectivity in Nigeria in 2002 facilitates the use of this technology for education and health promotion interventions aimed at adolescents. For example, short text messages on mobile phones have been successfully used to deliver reproductive health messages to female high school students in Ibadan [34]. High schools can be deemed an appropriate setting for health literacy education because many students are enrolled and can, therefore, be easily reached with peer-led interventions for consumer health literacy.

We found that after the training, the PEs in our project had improved their knowledge of consumer health literacy and their understanding of their roles as PEs. This was likely to have a positive impact on adolescents’ health literacy, as they had access to health information online. This finding is similar to the results of previous studies among high school students showing that their health knowledge improved after a well-planned educational intervention [34, 36]. For example, an intervention using sign language improved knowledge about HIV/AIDS among deaf high school students in Ibadan [36]. This improvement is particularly desirable in a peer education project when PEs are not only expected to be knowledgeable, but are also required to inform, counsel, and educate others. The improvement in knowledge can be attributed to trainers facilitating experiences and hands-on activities.

Data from the post-training interviews confirmed that PEs not only benefitted from the training, but also shared the new information they learned with others. Investigators who adopted a peer education approach for health promotion programs among adolescents have reported similar findings [37, 38]. Although their primary targets were fellow students, the PEs in our project extended their educational activities to other people, including their parents, outside the school environment. This is a major advantage of peer education, which is a strategy known to produce multiplier effects [37, 39].

Health discussions that PEs facilitated can also enhance the understanding of health information, which is likely to lead to behavior change [40]. There are three inherent merits of the peer education strategy when adopted in adolescent health programming. First, adolescents know better than adults about how to talk to their peers and motivate them to act, especially for sensitive issues like reproductive health, which are highly influenced by peer pressure [41]. Second, PEs can reach their colleagues whenever and wherever health literacy topics arise: inside the classrooms, on the school campus, at home, on street corners, and in bus stations. Third, trained adolescents derive long-term benefits from developing a sense of belonging and leadership skills [42]. Peer education also happens when students work together on a common task and have a chance to share evidence of their efforts through social media [43].

We acknowledge some limitations of our project. We do not know the number of persons the PEs reached and do not have information on how those they reached applied the knowledge they received from the PEs. Future programming should address this shortcoming as part of an effort to ensure the sustainability of health literacy interventions in schools. Also, approximately 28.0% of PEs in our project did not submit any activity form within the 4 months after training. Although non-submission of the forms did not necessarily imply nonperformance of the CHI activities, there was no other means of verifying the PEs’ activities. This challenge is not completely new, as attrition is a major problem of peer education interventions [32, 37]. Nevertheless, this project demonstrates the value of collaboration between librarians and other stakeholders in implementing a CHI intervention to address needs of in-school adolescents in Nigeria.

## SUPPLEMENTAL FILES

Appendix A Leaflet

Appendix B Consumer health information literacy project pre-post test questionnaire

Appendix C Peer education activity form

Appendix D Interview guide
