# Supplementary material for: Teaching high school students to use online consumer health resources on mobile phones: outcome of a pilot project in Oyo State, Nigeria
Source: J Med Libr Assoc. 2019 Apr 1;107(2):194–202. doi: 10.5195/jmla.2019.536 (PMC6466491; doi:10.5195/jmla.2019.536)

JMLA Journal of the Medical Library Association

SC
Supplemental content to
J Med Libr Assoc. Apr;107(2):dx.doi.org/10.5195/jmla.2019.536
www.jmla.mlanet.org
© Ajuwon, Ajuwon 2019

Teaching high school students to use online consumer health resources on mobile phones: outcome of a pilot project in Oyo State, Nigeria

Grace Ada Ajuwon; Ademola Johnson Ajuwon

APPENDIX A

Leaflet

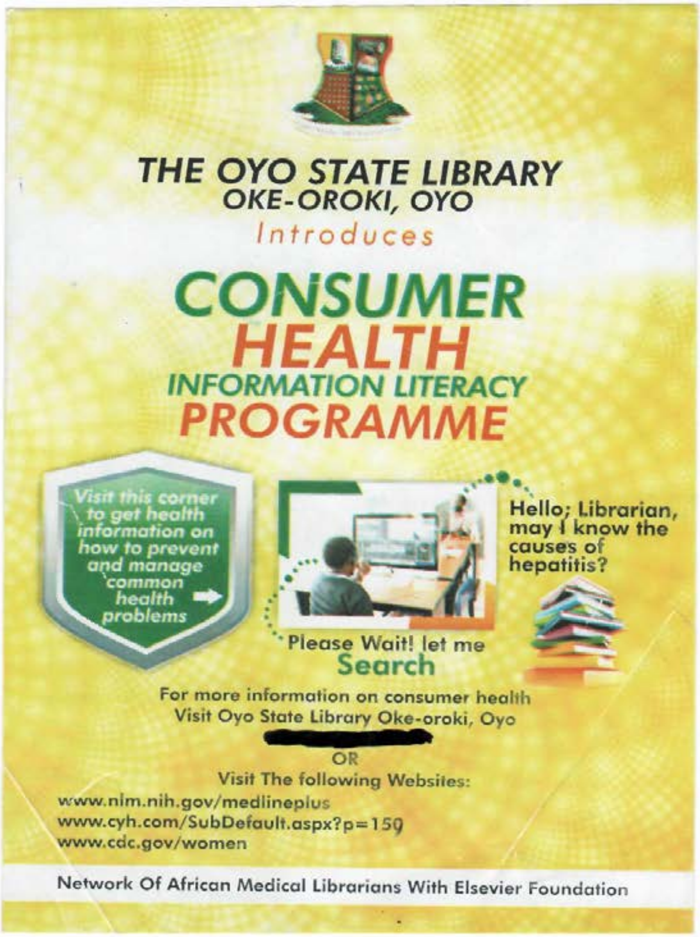

Supplement: Appendix A [file jmla-107-194-s001.pdf]
