# Supplementary material for: Teaching high school students to use online consumer health resources on mobile phones: outcome of a pilot project in Oyo State, Nigeria
Source: J Med Libr Assoc. 2019 Apr 1;107(2):194–202. doi: 10.5195/jmla.2019.536 (PMC6466491; doi:10.5195/jmla.2019.536)
Supplement: Appendix B [file jmla-107-194-s002.pdf]

JMLA Journal of the Medical Library Association

SC

Supplemental content to
J Med Libr Assoc. Apr;107(2):dx.doi.org/10.5195/jmla.2019.536
www.jmla.mlanet.org
© Ajuwon, Ajuwon 2019

Teaching high school students to use online consumer health resources on mobile phones: outcome of a pilot project in Oyo State, Nigeria

Grace Ada Ajuwon; Ademola Johnson Ajuwon

APPENDIX B

Consumer health information literacy project pre-post test questionnaire

A. Demographic profiles of students

1. Telephone number: ____

2. Name of school: ____

3. Age: ____________ years 4. Gender: 1. Male 2. Female

5. Class: 1. SS1 2. SS2 6. Date: ________________________

6. Religion: 1. Christianity 2. Islam 3. Other ______________________ (specify)

B. Knowledge of online health information

1. Have you ever used your phone to browse the Internet 1. Yes 2. No
2. Have you ever heard of consumer health information? 1. Yes 2. No
3. List all sources of information on health that you know

a. ____ b. ____

c. ____ d. ____

4. List websites where students can get health information from the Internet

a. ____ b. ____

c. ____ d. ____

5. Which site did you visit on the Internet for health information?
   1. Yahoo 2. Google 3. Facebook 4. TeensHealth
6. Define who a peer educator is ____
7. List characteristics of a good peer educator ____
