# Supplementary material for: Teaching high school students to use online consumer health resources on mobile phones: outcome of a pilot project in Oyo State, Nigeria
Source: J Med Libr Assoc. 2019 Apr 1;107(2):194–202. doi: 10.5195/jmla.2019.536 (PMC6466491; doi:10.5195/jmla.2019.536)
Supplement: Appendix D [file jmla-107-194-s004.pdf]

JMLA Journal of the Medical Library Association

SC

Supplemental content to
J Med Libr Assoc. Apr;107(2):dx.doi.org/10.5195/jmla.2019.536
www.jmla.mlanet.org
© Ajuwon, Ajuwon 2019

Teaching high school students to use online consumer health resources on mobile phones: outcome of a pilot project in Oyo State, Nigeria

Grace Ada Ajuwon; Ademola Johnson Ajuwon

APPENDIX D

Interview guide

Consumer Health Information Project

Introduction:

Welcome: Welcome and thank you for agreeing to participate in this discussion. We appreciate your willingness to participate.

Your name: My name is __________________ and I will be moderating this discussion.

The name of my partner is _________________ and he will be taking notes of our discussion today.

Purpose: I/We are here to conduct discussions with you based on the training you received on consumer health information literacy sometime this year. The reason we are having the discussions is to describe how you have used the training you received to improve your health literacy and those of people around you. We are also interested in finding out how the training has been of benefit to you and how it can be improved. We want to learn from you so we will be happy if you share your honest and open thoughts with us.

Confidentiality: What is said in this room stays here. I want you to feel comfortable sharing your experiences and thoughts. Although we will be taking some notes during the session, we can't possibly write fast enough to get it all down, so we would like to record our conversation on audiotape to enable us capture everything we discuss. We will not identify you by name in our report. Your name and your responses will remain anonymous. The information you provide will be a secret.

Duration: The interview should take at most an hour.

How the interview will be conducted: There are no right or wrong answers. Your experiences and opinions are important, so please speak freely during this discussion. Remember, you don't have to talk about anything you don't want to and you may end the discussion at any time.

Opportunity for questions: Are there any question about what I have just explained? Are you willing to participate in this discussion? Yes No

Consent of participants: I thank you for agreeing to participate in the study.

1. In what way has the training been useful to you?
2. How have you done the work of teaching your peers how to find health information?
3. Which of the activities in the form have you been doing?
4. Which of the activities in the form was most difficult for you to do?
5. When was the last time you talked to your friend or classmate on how she/he can access health information on the Internet?
6. What health information did you provide to your friends/classmates/schoolmates?
7. How did your friends/classmates receive the information you provided to them?

JMLA Journal of the Medical Library Association

Supplemental content to
J Med Libr Assoc. Apr;107(2):dx.doi.org/10.5195/jmla.2019.536
www.jmla.mlanet.org
© Ajuwon, Ajuwon 2019

8. How did you benefit from the training program?
9. How can the program be improved?
10. What would you want to be included in or removed from the training program?
